# A Tensor-Product-Kernel Framework for Multiscale Neural Activity Decoding and Control

**DOI:** 10.1155/2014/870160

**Published:** 2014-04-14

**Authors:** Lin Li, Austin J. Brockmeier, John S. Choi, Joseph T. Francis, Justin C. Sanchez, José C. Príncipe

**Affiliations:** ^1^Philips Research North America, Briarcliff Manor, NY 10510, USA; ^2^Department of Electrical and Computer Engineering, University of Florida, Gainesville, FL 32611, USA; ^3^Joint Program in Biomedical Engineering, NYU Polytechnic School of Engineering and SUNY Downstate, Brooklyn, NY 11203, USA; ^4^Department of Physiology and Pharmacology, State University of New York Downstate Medical Center, Joint Program in Biomedical Engineering, NYU Polytechnic School of Engineering and SUNY Downstate, Robert F. Furchgott Center for Neural & Behavioral Science, Brooklyn, NY 11203, USA; ^5^Department of Biomedical Engineering, Department of Neuroscience, Miami Project to Cure Paralysis, University of Miami, Coral Gables, FL 33146, USA

## Abstract

Brain machine interfaces (BMIs) have attracted intense attention as a promising technology for directly interfacing computers or prostheses with the brain's motor and sensory areas, thereby bypassing the body. The availability of multiscale neural recordings including spike trains and local field potentials (LFPs) brings potential opportunities to enhance computational modeling by enriching the characterization of the neural system state. However, heterogeneity on data type (spike timing versus continuous amplitude signals) and spatiotemporal scale complicates the model integration of multiscale neural activity. In this paper, we propose a tensor-product-kernel-based framework to integrate the multiscale activity and exploit the complementary information available in multiscale neural activity. This provides a common mathematical framework for incorporating signals from different domains. The approach is applied to the problem of neural decoding and control. For neural decoding, the framework is able to identify the nonlinear functional relationship between the multiscale neural responses and the stimuli using general purpose kernel adaptive filtering. In a sensory stimulation experiment, the tensor-product-kernel decoder outperforms decoders that use only a single neural data type. In addition, an adaptive inverse controller for delivering electrical microstimulation patterns that utilizes the tensor-product kernel achieves promising results in emulating the responses to natural stimulation.

## 1. Introduction

Brain machine interfaces (BMIs) provide new means to communicate with the brain by directly accessing, interpreting, and even controlling neural states. They have attracted attention as a promising technology to aid the disabled (i.e., spinal cord injury, movement disability, stroke, hearing loss, and blindness) [1–6]. When designing neural prosthetics and brain machine interfaces (BMIs), the fundamental steps involve quantifying the information contained in neural activity, modeling the neural system, decoding the intention of movement or stimulation, and controlling the spatiotemporal neural activity pattern to emulate natural stimulation. Furthermore, the complexity and distributed dynamic nature of the neural system pose challenges for the modeling tasks.

The development of recording technology enables access to brain activity from multiple functional levels, including the activity of individual neurons (spike trains), local field potentials (LFPs), electrocorticogram (ECoG), and electroencephalogram (EEG), collectively forming a multiscale characterization of brain state. Simultaneous recording of multiple types of signals could facilitate enhanced neural system modeling. Although there are underlying relationships among these brain activities, it is unknown how to leverage the heterogeneous set of signals to improve the identification of the neural-response-stimulus mappings. The challenge is in defining a framework that can incorporate these heterogenous signal formats coming from multiple spatiotemporal scales. In our work, we mainly address integrating spike trains and LFPs for multiscale neural decoding.

Spike trains and LFPs encode complementary information of stimuli and behaviors [7, 8]. In most recordings, spike trains are obtained by detecting transient events on a signal that is conditioned using a high-pass filter with the cutoff frequency set at about 300–500 Hz, while LFPs are obtained by using a low-pass filter with 300 Hz cutoff frequency [9]. Spike trains represent single- or multiunit neural activity with a fine temporal resolution. However, their stochastic properties induce considerable variability, especially when the stimulation amplitude is small; that is, the same stimuli rarely elicit the same firing patterns in repeated trials. In addition, a functional unit of the brain contains thousands of neurons. Only the activity of a small subset of neurons can be recorded and, of these, only a subset may modulate with respect to the stimuli or condition of interest.

In contrast, LFPs reflect the average synaptic input to a region near the electrode [10], which limits specificity but provides robustness for characterizing the modulation induced by stimuli. Furthermore, LFPs naturally provide population-level measure of neural activity. Therefore, an appropriate aggregation of LFPs and spike trains enables enhanced accuracy and robustness of neural decoding models. For example, the decoder can coordinate LFPs or spike patterns to tag particularly salient events or extract different stimulus features characterized by multisource signals. However, heterogeneity between LFPs and spike trains complicates their integration into the same model. The information in a spike train is coded in a set of ordered spike timings [11, 12], while an LFP is a continuous amplitude time series. Moreover, the time scale of LFPs is significantly longer than spike trains. Whereas recent work has compared the decoding accuracy of LFPs and spikes [13], only a small number of simple models have been developed to relate both activities [14]. However, the complete relationship between LFPs and spike trains is still a subject of controversy [15–17], which hinders principled modeling approaches.

To address these modeling issues, this paper proposes a signal processing framework based on tensor-product kernels to enable decoding and even controlling multiscale neural activities. The tensor-product kernel uses multiple heterogenous signals and implicitly defines a kernel space constructed by the tensor product of individual kernels designed for each signal type [18]. The tensor-product kernel uses the joint features of spike trains and LFPs. This enables kernel-based machine learning methodologies to leverage multiscale neural activity to uncover the mapping from the neural system states and the corresponding stimuli.

The kernel least mean square (KLMS) algorithm is used to estimate the dynamic nonlinear mapping from the two types of neural responses to the stimuli. The KLMS algorithm exploits the fact that the linear signal processing in reproducing kernel Hilbert spaces (RKHS) corresponds to nonlinear processing in the input space and is used in the adaptive inverse control scheme [19] designed for controlling neural systems. Utilizing the tensor-product kernel, we naturally extend this scheme to multiscale neural activity. Since the nonlinear control is achieved via linear processing in the RKHS, it bypasses the local minimum issues normally encountered in nonlinear control.

The validation of the effectiveness of the proposed tensor-product-kernel framework is done in a somatosensory stimulation study. Somatosensory feedback remains underdeveloped in BMI, which is important for motor and sensory integration during movement execution, such as proprioceptive and tactile feedback about limb state during interaction with external objects [20, 21]. A number of early experiments have shown that spatiotemporally patterned microstimulation delivered to somatosensory cortex can be used to guide the direction of reaching movements [22–24]. In order to effectively apply the artificial sensory feedback in BMI, it is essential to find out how to use microstimulation to replicate the target spatiotemporal patterns in the somatosensory cortex, where neural decoding and control are the critical technologies to achieve this goal. In this paper, our framework is applied to leverage multiscale neural activities to decode both natural sensory stimulation and microstimulation. Its decoding accuracy is compared with decoders that use a single type of neural activity (LFPs or spike trains).

In the neural system control scenario, this tensor-product-kernel methodology can also improve the controller performance. Controlling the neural activity via stimulation has raised the prospect of generating specific neural activity patterns in downstream areas, even mimicking natural neural responses, which is central both for our basic understanding of neural information processing and for engineering “neural prosthetic” devices that can interact with the brain directly [25]. From a control theory perspective, the neural circuit is treated as the “plant,” where the applied microstimulation is the control signal and the plant output is the elicited neural response represented by spike trains and LFPs. Most conventional control schema cannot be directly applied to spike trains because there is no algebraic structure in the space of spike trains. Therefore, most existing neural control approaches have been applied to binned spike trains or LFPs [25–31]. Here, we will utilize the kernel-based adaptive inverse controller for spike trains proposed in our previous work [19] as an input-output (system identification) based control scheme. This methodology can directly be extended to the tensor-product kernel to leverage the availability of multiscale neural signals (e.g., spike trains and LFPs) and improves the robustness and accuracy of the stimulation optimization by exploiting the complementary information of the heterogenous neural signals recorded from multiple sources.

The adaptive inverse control framework controls patterned electrical microstimulation in order to drive neural responses to mimic the spatiotemporal neural activity patterns induced by tactile stimulation. This framework creates new opportunities to improve the ability to control neural states to emulate the natural stimuli by leveraging the complementary information from multiscale neural activities. This better interprets the neural system internal states and thus enhances the robustness and accuracy of the optimal microstimulation pattern estimation.

The rest of the paper is organized as follows.  Section 2 introduces kernels for spike trains and LFPs and the tensor-product kernel that combines them. The kernel-based decoding model and the adaptive inverse control scheme that exploit kernel-based neural decoding technology to enable control in RKHS are introduced in Sections 3 and 4, respectively.  Section 5 discusses the somatosensory stimulation emulation scenario and illustrates the test results by applying tensor-product kernel to leverage multiscale neural activity for decoding and controlling tasks.  Section 6 concludes this paper.

## 2. Tensor-Product Kernel for Multiscale Heterogeneous Neural Activity

The mathematics of many signal processing and pattern recognition algorithms is based on evaluating the similarity of pairs of exemplars. For vectors or functions, the inner product defined on Hilbert spaces is a linear operator and a measure of similarity. However, not all data types exist in Hilbert spaces. Kernel functions are bivariate, symmetric functions that implicitly embed samples in a Hilbert space. Consequently, if a kernel on a data type can be defined, then algorithms defined in terms of inner products can be used. This has enabled various kernel algorithms [18, 32–34].

To begin, we define the general framework for the various kernel functions used here, keeping in mind that the input corresponds to assorted neural data types. Let the domain of a single neural response dimension, that is, a single LFP channel or one spiking unit, be denoted by *𝒳* and consider a kernel *κ* : *𝒳* × *𝒳* → ℝ. If *κ* is positive definite, then there is an implicit mapping *ϕ* : *𝒳* → *ℋ* that maps any two sample points, say *x*, *x*′ ∈ *𝒳*, to corresponding elements in the Hilbert space *ϕ*(*x*), *ϕ*(*x*′) ∈ *ℋ* such that *κ*(*x*, *x*′) = 〈*ϕ*(*x*), *ϕ*(*x*′)〉 is the inner product of these elements in the Hilbert space. As an inner product, the kernel evaluation *κ*(*x*, *x*′) quantifies the similarity between *x* and *x*′.

A useful property is that this inner product induces a distance metric,

(1) d(x,x′)=||ϕ(x)−ϕ(x′)||ℋ=〈ϕ(x)−ϕ(x′),ϕ(x)−ϕ(x′)〉=κ(x,x)+κ(x′,x′)−2κ(x,x′).

For normalized and shift-invariant kernels where, for all *x*, *κ*(*x*, *x*) = 1, the distance is inversely proportional to the kernel evaluation $d(x,x^{\prime })=\sqrt{2-2\kappa (x,x^{\prime })}$.

To utilize two or more dimensions of the neural response, a kernel that operates on the joint space is required. There are two basic approaches to construct multidimensional kernels from kernels defined on the individual variables: direct sum and tensor-product kernels. In terms of kernel evaluations, they consist of taking either the sum or the product of the individual kernel evaluations. In both cases, the resulting kernel is positive definite as long as the individual kernels are positive definite [18, 35].

Let *𝒳*_*i*_ denote the neural response domain of the *i*th dimension and consider a positive-definite kernel *κ*_*i*_ : *𝒳*_*i*_ × *𝒳*_*i*_ → ℝ and corresponding mapping *ϕ*_*i*_ : *𝒳*_*i*_ → *ℋ*_*i*_ for this dimension. Again, the similarity between a pair of samples *x* and *x*′ on the *i*th dimension is *κ*_*i*_(*x*_(*i*)_, *x*_(*i*)_′) = 〈*ϕ*_*i*_(*x*_(*i*)_), *ϕ*_*i*_(*x*_(*i*)_′)〉.

For the sum kernel, the joint similarity over a set of dimensions *ℐ* is

(2) $$\begin{array}{c} \kappa_{\Sigma }\left(x,x^{\prime }\right)=\underset{i\in ℐ}{\sum }\kappa_{i}\left(x_{\left(i\right)},x_{\left(i\right)}^{\prime }\right). \end{array}$$

This measure of similarity is an average similarity across all dimensions. When the sum is over a large number of dimensions, the contributions of individual dimensions are diluted. This is useful for multiunit spike trains or multichannel LFPs when the individual dimensions are highly variable, which if used individually would result in a poor decoding performance on a single trial basis.

For the tensor-product kernel, the joint similarity over two dimensions *i* and *j* is computed by taking the product between the kernel evaluations *κ*_[*i*,*j*]_([*x*_(*i*)_, *x*_(*j*)_], [*x*_(*i*)_′, *x*_(*j*)_′]) = *κ*_*i*_(*x*_(*i*)_, *x*_(*i*)_′) · *κ*_*j*_(*x*_(*j*)_, *x*_(*j*)_′). The new kernel *κ*_[*i*,*j*]_ corresponds to a mapping function that is the tensor product between the individual mapping functions *ϕ*_[*i*,*j*]_ = *ϕ*_*i*_ ⊗ *ϕ*_*j*_ where *ϕ*_[*ij*]_(*x*_(*i*,*j*)_) ∈ *ℋ*_[*ij*]_. This is the tensor-product Hilbert space. The product can be taken over a set of dimensions *ℐ* and the result is a positive-definite kernel over the joint space: *κ*_Π_(**x**, **x**′) = ∏_*i*∈*ℐ*_ *κ*_*i*_(*x*_(*i*)_, *x*_(*i*)_′).

The tensor-product kernel corresponds to a stricter measure of similarity than the sum kernel. Due to the product, if for one dimension *κ*_*i*_(*x*_(*i*)_, *x*_(*i*)_′) ≈ 0 then *κ*_Π_(**x**, **x**′) ≈ 0. The tensor-product kernel requires the joint similarity; that is, for samples to be considered similar in the joint space they must be close in all the individual spaces. If even one dimension is dissimilar the product will appear dissimilar. If some of the dimensions are highly variable, then they will have a deleterious effect on the joint similarity measure. On the other hand, the tensor product is a more precise measure of similarity that will be used later to combine multiscale neural activity.

More generally, an explicit weight can be used to adjust the influence of the individual dimensions on the joint kernel. Any convex combinations of kernels are positive definite, and learning the weights of this combination is known as multiple kernel learning [36–38]. In certain cases of the constituent kernels, namely, that they are infinitely divisible [39], a weighted product kernel can also be applied [40]. However, the optimization of these weightings is not explored in the current work.

**Figure 1:**
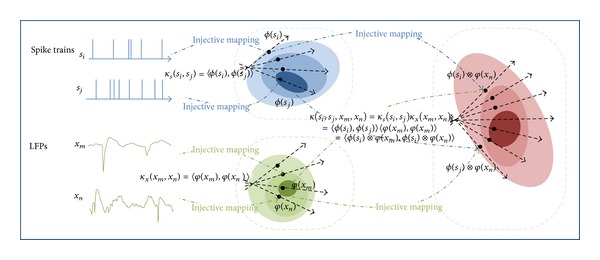
Schematic representation of the construction of the RKHS defined by the tensor-product kernel from the individual spike and LFP kernel spaces, along with the mapping from the original data. Specifically, **s**_*i*_ denotes a window of multiunit spike trains; **x**_*n*_ denotes a window of multichannel LFPs; *κ*_*s*_(·, ·) denotes the spike train kernel with implicit mapping function *ϕ*(·); and *κ*_*x*_(·, ·) denotes the LFP kernel with implicit mapping function *φ*(·).

In general, a joint kernel space, constructed via either direct sum or tensor product, allows the homogeneous processing of heterogenous signal types all within the framework of RKHSs. We use direct sum kernels to combine the different dimensions of multiunit spike trains or multichannel LFPs. For the spike trains, using the sum kernel across the different units enables an “average” population similarity over the space of spike trains where averages cannot be computed. Then a tensor-product kernel combines the two kernels: one for the multiunit spike trains and one for the multichannel LPFs; see Figure 1 for an illustration. The kernels for spike trains and LFPs can be selected and specified individually according to their specific properties.

In conclusion, composite kernels are very different from those commonly used in kernel-based machine learning, for example, for the support vector machine. In fact, here a pair of windowed spike trains and windowed LFPs is mapped into a feature function in the joint RKHS. Different spike train and LFP pairs are mapped to different locations in this RKHS, as shown in Figure 1. Due to its versatility, Schoenberg kernels defined for both the spike timing space and LFPs are employed in this paper and discussed below.

### 2.1. Kernel for Spike Trains

Unlike conventional amplitude data, there is no natural algebraic structure in the space of spike trains. The binning process, which easily transforms the point processes into discrete amplitude time series, is widely used in spike train analysis and allows the application of conventional amplitude-based kernels to spike trains [41] at the expense of losing the temporal resolution of the neural responses. This means that any temporal information in spikes within and between bins is disregarded, which is especially alarming when spike timing precision can be in the millisecond range. Although the bin size can be set small enough to preserve the fine time resolution, it will sparsify the signal representation, increase the artifact variability, and cause high-dimensionality in the model, which requires voluminous data for proper training.

According to the literature, it is appropriate to consider a spike train to be a realization of a point process, which describes the temporal distribution of the spikes. Generally speaking, a point process *p*_*i*_ can be completely characterized by its conditional intensity function *λ*(*t* | *H*_*t*_^*i*^), where *t* ∈ *τ* = [0, *T*] denotes the time coordinate and *H*_*t*_^*i*^ is the history of the process up to *t*. A recent research area [42, 43] is to define an injective mapping from spike trains to RKHS based on the kernel between the conditional intensity functions of two point processes [42]. Among the cross-intensity (CI) kernels, the Schoenberg kernel is defined as

(3) κ(λ(t ∣ Hti),λ(t ∣ Htj)) =exp⁡(−||λ(t ∣ Hti)−λ(t ∣ Htj)||2σ2) =exp⁡(−∫τ(λ(t ∣ Hti)−λ(t ∣ Htj))2dtσ2),

where *σ* is the kernel size. The Schoenberg kernel is selected in this work because of its modeling accuracy and robustness to free parameter settings [44]. The Schoenberg kernel is a Gaussian-like kernel defined on intensity functions that is strictly positive definite and sensitive to the nonlinear coupling of two intensity functions [42]. Different spike trains will then be mapped to different locations in the RKHS. Compared to kernels designed on binned spike trains (e.g., spikernel [41]), the main advantage of the Schoenberg kernel is that the precision in the spike event location is better preserved and the limitations of the sparseness and high-dimensionality for model building are also avoided, resulting in enhanced robustness and reduced computational complexity, especially when the application requires fine time resolution [44].

In order to be applicable, the methodology must lead to a simple estimation of the quantities of interest (e.g., the kernel) from experimental data. A practical choice used in our work estimates the conditional intensity function using a kernel smoothing approach [42, 45], which allows estimating the intensity function from a single realization and nonparametrically and injectively maps a windowed spike train into a continuous function. The estimated intensity function is obtained by simply convolving *s*(*t*) with the smoothing kernel *g*(*t*), yielding

(4) $$\begin{array}{c} \hat{\lambda }\left(t\right)=\overset{M}{\underset{m=1}{\sum }}g\left(t-t_{m}\right),\;\left\{t_{m}\in 𝒯:m=1,\ldots ,M\right\}, \end{array}$$

where the smoothing function *g*(*t*) integrates to 1. Here $\hat{\lambda }(t)$ can be interpreted as an estimation of the intensity function. The rectangular and exponential functions [42, 46] are both popular smoothing kernels, which guarantee injective mappings from the spike train to the estimated intensity function. In order to decrease the kernel computation complexity, the rectangular function *g*(*t*) = (1/*𝒯*)(*U*(*t*) − *U*(*t* − *𝒯*)) (*𝒯*≫ the interspike interval) is used in our work, where *U*(*t*) is a Heaviside function. This rectangular function approximates the cumulative density function of spikes counts in the window *T* and compromises the locality of the spike trains; that is, the mapping places more emphasis on the early spikes than the later ones. However, our experiments show that this compromise only causes a minimal impact on the kernel-based regression performance.

Let *s*_*i*_^*n*^(*t*) denote the spike train for the *i*th sample of the *n*th spiking unit. The multiunit spike kernel is taken as the unweighted sum over the kernels on the individual units

(5) $$\begin{array}{c} \kappa_{s}\left(s_{i}\left(t\right),s_{j}\left(t\right)\right)=\underset{n}{\sum }\kappa_{s}\left(s_{i}^{n}\left(t\right),s_{j}^{n}\left(t\right)\right). \end{array}$$

### 2.2. Kernels for LFPs

In contrast with spike trains, LFPs exhibit less spatial and temporal selectivity [15]. In the time domain, LFP features can be obtained by sliding a window on the signal, which describes the temporal LFP structure. The length of the window is selected based on the duration of neural responses to certain stimuli; the extent of the duration can be assessed by its autocorrelation function, as we will discuss in Section 5.3.1. In the frequency domain, the spectral power and phase in different frequency bands are also known to be informative features for decoding, but here we concentrate only on the time-domain decompositions. In the time domain, we can simply treat single channel LFPs as a time series and apply the standard Schoenberg kernel to the sequence of signal samples in time. The Schoenberg kernel, defined in continuous spaces, maps the correlation time structure of the LFP *x*(*t*) into a function in RKHS,

(6) κx(xi(t),xj(t)) =exp⁡(−||xi(t)−xj(t)||2σx2) =exp⁡(−∫𝒯x(xi(t)−xj(t))2dtσx2) =exp⁡(−(∫𝒯xxi(t)xi(t)          +xj(t)xj(t)−2xi(t)xj(t)dt)        × (σx2)−1),

where *𝒯*_*x*_ = [0  *T*_*x*_].

Let *x*_*i*_^*n*^(*t*) denote the LFP waveform for the *i*th sample of the *n*th channel. Similar to the multiunit spike kernel, the multichannel LFP kernel is defined by the direct sum kernel

(7) $$\begin{array}{c} \kappa_{x}\left(x_{i}\left(t\right),x_{j}\left(t\right)\right)=\underset{n}{\sum }\kappa_{x}\left(x_{i}^{n}\left(t\right),x_{j}^{n}\left(t\right)\right). \end{array}$$

### 2.3. Discrete Time Sampling

Assuming a sampling rate with period *τ*, let **x**_*i*_ = [*x*_*i*_^1^, *x*_*i*+1_^1^,…, *x*_*i*−1+*T*/*τ*_^1^, *x*_*i*_^2^,…, *x*_*i*−1+*T*/*τ*_^*N*^] denote the *i*th multichannel LFP vector obtained by sliding the *T*-length window with step *τ*. Let **s**_*i*_ = {*t*_*m*_ − (*i* − 1)*τ*, *t*_*m*_ ∈ [(*i* − 1)*τ*, (*i* − 1)*τ* + *T*] : *m* = 1,…, *M*} denote the corresponding *i*th window of the multiunit spike timing sequence. The time scale, both in terms of the window length and sampling rate, of the analysis for LFPs and spikes is very important and needs to be defined by the characteristics of each signal. The tensor-product kernel allows the time scales of the analysis for LFPs and spike trains to be specified individually; that is, the window length *T* and sampling rate for spike trains and LFPs could be different. The suitable time scale can be estimated through autocorrelation coefficients of the signal as will be explained below.

## 3. Adaptive Neural Decoding Model

For neural decoding applications, a regression model with multiscale neural activities as the input is built to reconstruct a stimulus. The appeal of kernel-based filters is the usage of the linear structure of RKHS to implement well-established linear adaptive algorithms and to obtain a nonlinear filter in the input space that leads to universal approximation capability without the problem of local minima. There are several candidate kernel-based regression methods [32], such as support vector regression (SVR) [33], kernel recursive least squares (KRLS), and kernel least mean square (KLMS) [34]. The KLMS algorithm is preferred here because it is an online methodology of low computation complexity.

The quantized kernel least mean square (Q-KLMS) is selected in our work to decrease the filter growth.  Algorithm 1 shows the pseudocode for the Q-KLMS algorithm with a simple online vector quantization (VQ) method, where the quantization is performed based on the distance between the new input and each existing center. In this work, this distance between a center and the input is defined by their distance in RKHS, which for a shift-invariant normalized kernel for all *xκ*(*x*, *x*) = 1 is ||*ϕ*(*x*_*n*_)−*ϕ*(*x*_*i*_)||_2_^2^ = 2 − 2*κ*(*x*_*n*_, *x*_*i*_). If the smallest distance is less than a prespecified quantization size *ɛ*, the new coefficient *ηe*_*n*_ adjusts the weight of the closest center; otherwise a new center is added. Compared to other techniques [47–50] that have been proposed to curb the growth of the networks, the simple online VQ method is not optimal but is very efficient. Since, in our work, the algorithm must be applied several times to the same data for convergence after the first iteration over the data, we choose *ɛ* = 0, which merges the repeated centers and enjoys the same performance as KLMS.

We use the Q-KLMS framework with the multiunit spike kernels, the multichannel LFP kernels, and the tensor-product kernel using the joint samples of LFPs and spike trains. This is quite unlike previous work in adaptive filtering that almost exclusively uses the Gaussian kernel with real-valued time series.

## 4. Adaptive Inverse Control of the Spatiotemporal Patterns of Neural Activity

**Figure 2:**
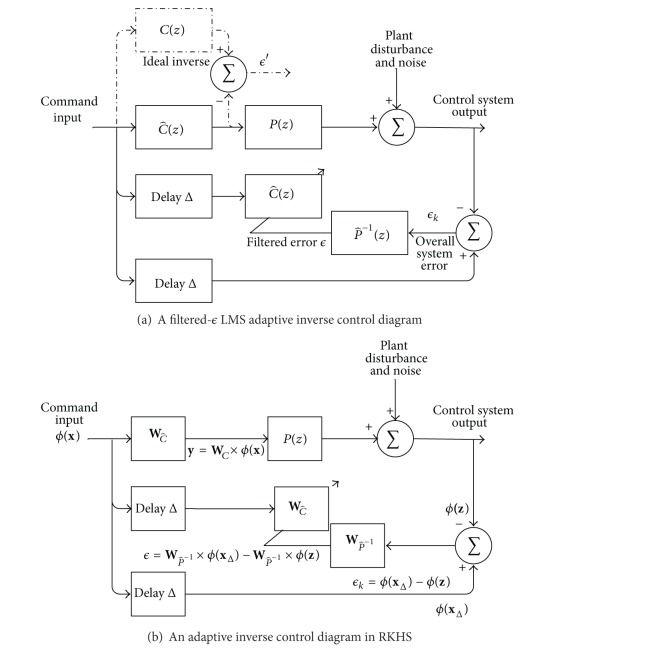
Adaptive inverse control diagram.

As the name indicates, the basic idea of adaptive inverse control is to learn an inverse model of the plant as the controller in Figure 2(a), such that the cascade of the controller and the plant will perform like a unitary transfer function, that is, a perfect wire with some delay. The target plant output is used as the controller's command input. The controller parameters are updated to minimize the dissimilarity between the target output and the plant's output during the control process, which enables the controller to track the plant variation and cancel system noises. The filtered-*ϵ* LMS adaptive inverse control diagram [51] shown in Figure 2(a) represents the filtered-*ϵ* approach to find $\hat{C}(z)$. If the ideal inverse controller *C*(*z*) is the actual inverse controller, the mean square of the overall system error *ϵ*_*k*_ would be minimized. The objective is to make $\hat{C}(z)$ as close as possible to the ideal *C*(*z*). The difference between the outputs of *C*(*z*) and $\hat{C}(z)$, both driven by the command input, is therefore an error signal *ϵ*′. Since the target stimulation is unknown, instead of *ϵ*′, a filtered error *ϵ*, obtained by filtering the overall system error *ϵ*_*k*_ through the inverse plant model ${\hat{P}}^{-1}(z)$, is used for adaptation in place of *ϵ*′.

If the plant has a long response time, a modeling delay is advantageous to capture the early stages of the response, which is determined by the sliding window length that is used to obtain the inverse controller input. There is no performance penalty from the delay Δ as long as the input to $\hat{C}(z)$ undergoes the same delay. The parameters of the inverse model ${\hat{P}}^{-1}(z)$ are initially modeled offline and updated during the whole system operation, which allows ${\hat{P}}^{-1}(z)$ to incrementally identify the inverse system and thus make *ϵ* approach *ϵ*′. Moreover, the adaptation enables ${\hat{P}}^{-1}(z)$ to track changes in the plant. Thus, minimizing the filter error obtained from ${\hat{P}}^{-1}(z)$ makes the controller follow the system variation.

### 4.1. Relation to Neural Decoding

In this control scheme, there are only two models, $\hat{C}(z)$ and ${\hat{P}}^{-1}(z)$, adjusted during the control process, which share the same input (neural activity) and output types (continuous stimulation waveforms). Therefore, both models perform like neural decoders and can be implemented using the Q-KLMS method we introduced in the previous section. Since all the mathematical models in this control scheme are kernel-based models, the whole control scheme can be mapped into an RKHS space, which holds several advantages as follows. (1) No restriction is imposed on the signal type. As long as a kernel can map the plant activity to RKHS, the plant can be controlled with this scheme. (2) Both the plant inverse, ${\hat{P}}^{-1}(z)$, and the controller $\hat{C}(z)$ have linear structure in RKHS, which avoids the danger of converging to local minima.

Specifically, the controller $\hat{C}(z)$ and the plant inverse ${\hat{P}}^{-1}(z)$ are separately modeled with the tensor-product kernel that we described in Section 2, and the model coefficients are updated with Q-KLMS. This structure is shown in Figure 2(b). The model coefficients $W_{\hat{C}}$ and $W_{{\hat{P}}^{-1}}$ represent the weight matrix of $\hat{C}(z)$ and ${\hat{P}}^{-1}(z)$ obtained by Q-KLMS, respectively. As this is a multiple-input multiple-output model, $W_{\hat{C}}$  and $W_{{\hat{P}}^{-1}}$ are the concatenation of the filter weights for each stimulation channel.

The variables **x**, **y**, and **z** denote the concatenation of the windowed target spike trains and LFPs as the command input of the controller, the estimated stimulation, and the plant output, respectively. **x**_Δ_ is delayed target signal, which is aligned with the plant output **z**. *ϕ*(·) represents the mapping function from input space to the RKHS associated with the tensor-product kernel.

The overall system error is defined as *ϵ*_*k*_ = *ϕ*(**x**_Δ_) − *ϕ*(**z**), which means that the controller's parameter adaptation seeks to minimize the distance in the RKHS between the target spike train/LFP and the output of the plant inverse ${\hat{P}}^{-1}(z)$. In this way, since the inverse model ${\hat{P}}^{-1}(z)$ has a linear structure in RKHS, the filtered error for stimulation channel *j* ∈ 1,…, *M* is

(8) $$\begin{array}{c} \epsilon \left(j\right)=W_{j}^{{\hat{P}}^{-1}}\phi \left(x_{\Delta }\right)-W_{j}^{{\hat{P}}^{-1}}\phi \left(z\right). \end{array}$$

The controller model $\hat{C}(z)$ has a single input **x**, corresponding to the concatenation of the spike trains and LFPs, and has an *M*-channel output **y**, corresponding to the microstimulation. Q-KLMS is used to model $\hat{C}(z)$ with *N* input samples. The target spike trains are repeated among different trials, which means that the repeated samples will be merged on the same kernel center of the first pass through the data by the quantization and thus the network size of the inverse controller is fixed (*N* centers). Only the coefficient matrix **a** is updated with the filtered error *ϵ* during the whole control operation. The output of $\hat{C}(z)$ can be calculated by

(9) [y1y2⋮yM]=[a11a12⋯a1Na12a22⋯a2N⋮⋮aM1aM2⋯aMN][ϕ(c1c^)′ϕ(c2c^)′⋮ϕ(cNc^)′]ϕ(x)=[a11a12⋯a1Na12a22⋯a2N⋮⋮aM1aM2⋯aMN][κ(c1c^,x)κ(c2c^,x)⋮κ(cNc^,x)],

where $c_{n}^{\hat{c}}$ is the *n*th center and *a*_*mn*_ is the coefficient assigned to the *n*th kernel center for the *m* channel of the output.

## 5. Sensory Stimulation Experiment

### 5.1. Experimental Motivation and Framework

We applied these methods to the problem of converting touch information to electrical stimulation in neural prostheses. Somatosensory information originating in the peripheral nervous system ascends through the ventral posterior lateral (VPL) nucleus of the thalamus on its way to the primary somatosensory cortex (S1). Since most cutaneous and proprioceptive information is relayed through this nucleus, we expect that a suitably designed electrode array could be used to selectively stimulate a local group of VPL neurons so as to convey similar information to cortex. Electrophysiological experiments [52] suggest that the rostral portion of the rat VPL nucleus carries a large amount of proprioceptive information, while the medial and caudal portions code mostly for cutaneous stimuli. Since the body maps for both VPL thalamus and S1 are known and fairly consistent, it is possible to implant electrode arrays in somatotopically overlapping areas of both regions.

**Figure 3:**
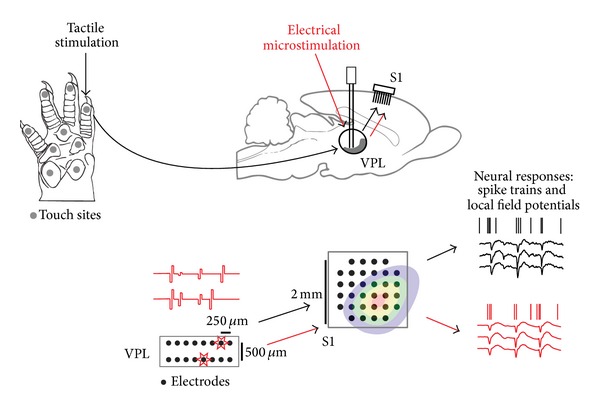
Neural elements in tactile stimulation experiments. To the left is the rat's hand with representative cutaneous receptive fields. When the tactor touches a particular “receptive field” on the hand, VPL thalamus receives this information and relays it to S1 cortex. To emulate “natural touch” with microstimulation, the optimized spatiotemporal microstimulus patterns are injected into the same receptive field on VPL thalamus through a microarray so that the target neural activity pattern can be replicated in somatosensory regions (S1) to convey the natural touch sensation to the animal.

We applied the proposed control method to generate multichannel electrical stimulation in VPL so as to evoke a naturalistic neural trajectory in S1.  Figure 3 shows a schematic depiction of our experiment, which was conducted in rats. After implanting arrays in both VPL and S1, the responses to natural stimulation, delivered by a motorized tactor, were recorded in S1. Then, we applied randomly patterned microstimulation in the VPL while recording the responses in S1. Using these responses, we then trained our controller to output the microstimulation patterns that would most accurately reproduce the neural responses to natural touch in S1. To solve this control problem, we first investigated how reliably the two types of stimulation, natural touch and electrical microstimulation, can be decoded.

### 5.2. Data Collection

All animal procedures were approved by the SUNY Downstate Medical Center IACUC and conformed to National Institutes of Health guidelines. A single female Long-Evans rat (Hilltop, Scottsdale, PA) was implanted with two microarrays while under anesthesia. After induction using isoflurane, urethane was used to maintain anesthetic depth. The array in VPL was a 2 × 8 grid of 70% platinum 30% iridium 75 *μ*m diameter microelectrodes (MicroProbes Inc.), with 500 *μ*m between the rows and 250 *μ*m interelectrode spacing within the rows. The microelectrodes had a 25 : 1 taper on the distal 5 mm with a tip diameter of 3 *μ*m. The approximate geometric surface area of the conducting tips was 1250 *μ*m^2^. The shank lengths were custom designed to fit the contour of the rat VPL [52]. Both rows were identical and the shaft lengths for each row, from medial to lateral, were (8,8, 8,8, 8,7.8,7.6,7.4) mm. The long axis of the VPL array was oriented along the rat's mediolateral axis.

The cortical electrode array (Blackrock Microsystems) was a 32-channel Utah array. The electrodes are arranged in a 6 × 6 grid excluding the 4 corners, and each electrode is 1.5 mm long. A single craniotomy that exposed the cortical insertions sites for both arrays was made, and, after several probing insertions with a single microelectrode (FHC) in an area 1 mm surrounding the stereotaxic coordinates for the digit region of S1 (4.0 mm lateral and 0.5 mm anterior to bregma) [53, 54], the Utah array was inserted using a pneumatic piston. The electrodes cover somatosensory areas of the S1 cortex and the VPL nucleus of the thalamus [52]. Neural recordings were made using a multichannel acquisition system (Tucker Davis).

Spike and field potential data were collected while the rat was maintained under anesthesia. The electrode voltages were preamplified with a gain of 1000, filtered with cutoffs at 0.7 Hz and 8.8 kHz, and digitized at 25 kHz. LFPs are further filtered from 1 to 300 Hz using a 3rd-order Butterworth filter. Spike sorting is achieved using *k*-means clustering of the first 2 principal components of the detected waveforms.

**Figure 4:**
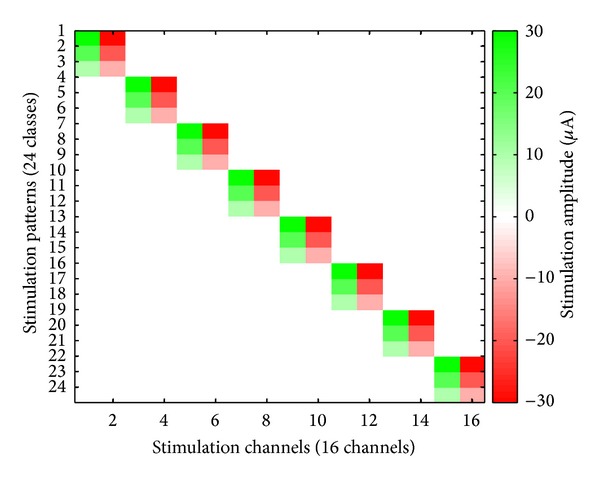
Bipolar microstimulation patterns applied in sensory stimulation experiment.

The experiment involves delivering microstimulation to VPL and tactile stimulation to the rat's fingers in separate sections. Microstimulation is administered on adjacent pairs (bipolar configurations) of the thalamic array. The stimulation waveforms are single symmetric biphasic rectangular current pulses; each rectangular pulse is 200 *μ*s long and has an amplitude of either 10 *μ*A, 20 *μ*A, or 30 *μ*A. Interstimulus intervals are exponentially distributed with mean interval of 100 ms. Stimulus isolation used a custom built switching headstage. The bipolar microstimulation pulses are delivered in the thalamus. There are 24 patterns of microstimulation: 8 different sites and 3 different amplitude levels for each site, as shown in Figure 4. Each pattern is delivered 125 times.

**Figure 5:**
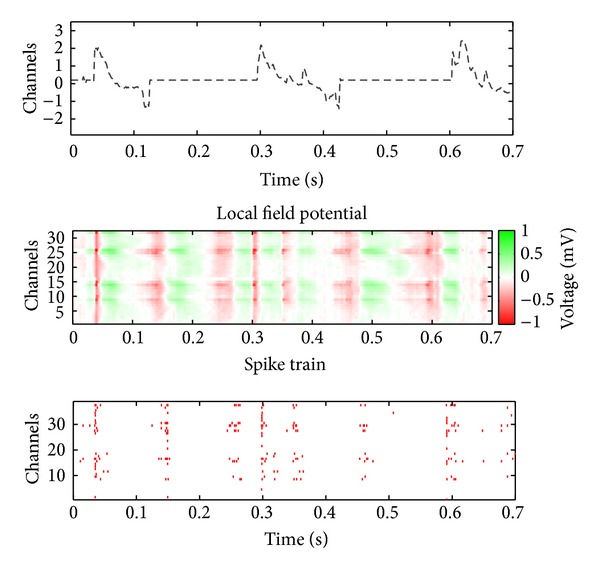
Rat neural response elicited by tactile stimulation. The upper plot shows the normalized derivative of tactile force. The remaining two plots show the corresponding LFPs and spike trains stimulated by tactile stimulation.

The experimental procedure also involves delivering 30–40 short 100 ms tactile touches to the rat's fingers (repeated for digit pads 1–4) using a hand-held probe. The rat remained anesthetized for the recording duration. The applied force is measured using a lever attached to the probe that pressed against a thin-film resistive force sensor (Trossen Robotics) when the probe tip contacted the rat's body. The resistive changes were converted to voltage using a bridge circuit and were filtered and digitized in the same way as described above. The digitized waveforms were filtered with a passband between 1 and 60 Hz using a 3rd-order Butterworth filter. The first derivative of this signal is used as the desired stimulation signal, which is shown in Figure 5.

### 5.3. Decoding Results

We now present the decoding results for the tactile stimulus waveform and microstimulation using Q-KLMS operating on the tensor-product kernel. The performance using the multiscale neural activity, both spike trains and LFPs, is compared with the decoder using single-type neural activity. This illustrates the effectiveness of the tensor-product-kernel-based framework to exploit the complementarity information from multiscale neural activities.

#### 5.3.1. Time Scale Estimation

The tensor-product kernel allows the time scales of the analysis for LFPs and spike trains to be specified individually, based on their own properties. In order to find reasonable time scales, we estimate the autocorrelation coefficients of LFPs and spike trains, which indicates the response duration induced by the stimulation. For this purpose, spike trains are binned with bin size of 1 ms. The LFPs are also resampled with sampling rate 1000 Hz. The autocorrelation coefficients of each signal average over channels are calculated by

(10) $$\begin{array}{c} {\hat{\rho }}_{h}=\frac{\sum_{t=h+1}^{T}\left(y_{t}-\bar{y}\right)\left(y_{t-h}-\bar{y}\right)}{\sum_{t=1}^{T}{\left(y_{t}-\bar{y}\right)}^{2}}. \end{array}$$

The 95% confidence bounds of the hypothesis that the autocorrelation coefficient is effectively zero are approximately estimated by ±2SE*ρ*, where

(11) $$\begin{array}{c} \text{SE}\rho =\sqrt{\frac{\left(1+2\sum_{i=1}^{h-1}\rho_{i}^{2}\right)}{N}}. \end{array}$$

**Figure 6:**
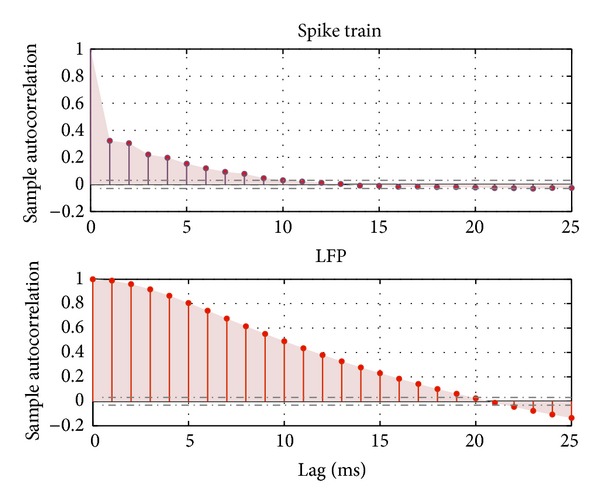
Autocorrelation of LFPs and spike trains for window size estimation.

The average confidence bounds for LFPs and spike trains are [−0.032  0.032] and [−0.031  0.031], respectively. The autocorrelation coefficients of LFPs fall into the confidence interval after 20 ms, while the autocorrelation coefficients of spike trains die out after 9 ms, as shown in Figure 6. Therefore, the decoder inputs are obtained by sliding the window with a size of *T*_*s*_ = 9 ms for spike trains and *T*_*x*_ = 20 ms for LFPs. In addition, the time discretization for the stimuli is 5 ms.

The learning rates for each decoder are determined by the best cross-validation results after scanning the parameters. The kernel sizes *σ*_*s*_ and *σ*_*x*_ are determined by the average distance in RKHS of each pair of training samples. The normalized mean square error (NMSE) between the estimated stimulus (**y**) and the desired stimulus (**d**) is utilized as an accuracy criterion.

#### 5.3.2. Results for Decoding Tactile Stimulation

NMSEs of tactile stimulation are obtained across 8 trial data sets. For each trial, we use 20 s data to train the decoders and compute an independent test error on the remaining 2.5 s data. The results are shown in Table 1, where we can observe that the LFP and spike decoder significantly outperformed both the LFP decoder and the spike decoder with *P* value <0.05.

**Figure 7:**
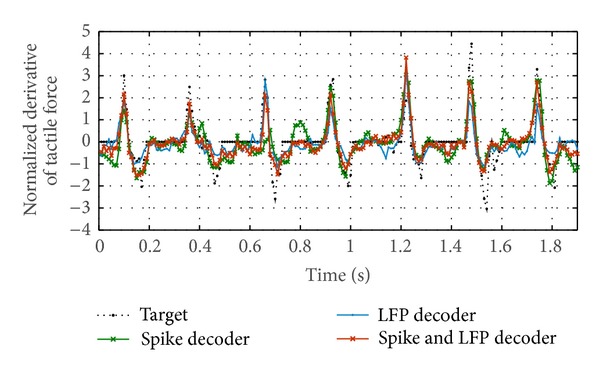
Qualitative comparison of decoding performance of the first tactile stimulation trial among LFP decoder, spike decoder, and spike and LFP decoder.

In order to illustrate the details of the decoding performance, a portion of the test results of the first trial are shown in Figure 7. It is observed that the output of the spike decoder fluctuates and misses some pulses (e.g., around 0.65 s) due to the sparsity and variability of spike trains. In contrast, the output estimated by LFP decoder is smooth and more robust than the spike decoder, but the decoded signal undershoots the maximum force deflections. The LFP and spike decoder performed better than the LFP decoder by reincorporating the precise pulse timing information from spike trains.

#### 5.3.3. Results for Decoding Electrical Microstimulation

We also implemented a decoder to reconstruct the microstimulation pattern. First, we mapped the 8 different stimulation configurations to 8 channels. We dismissed the shape of each stimulus, since the time scale of the stimulus width is only 200 *μ*s. The desired stimulation pattern of each channel is represented by a sparse time series of the stimulation amplitude.

**Figure 8:**
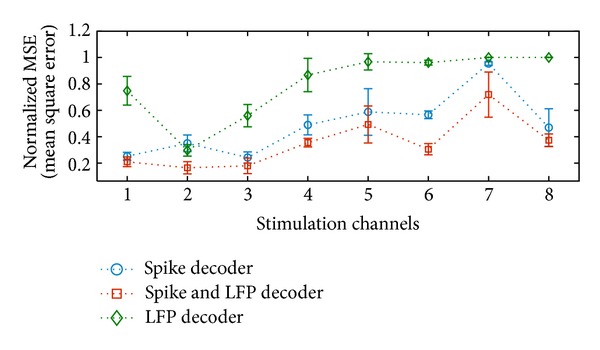
Performance comparison of the microstimulation reconstruction performance among spike decoder, LFP decoder, and spike and LFP decoder in terms of NMSE for each microstimulation channel.

NMSEs are obtained with ten subsequence decoding results. We used 120 s data to train the decoders and compute an independent test error on the remaining 20 s data. The spike and LFP decoder also outperformed both the LFP decoder and the spike decoder. The comparison of results is shown in Figure 8, which indicates that spike and LFP decoder is able to obtain the best performance amongst the stimulation channels, especially for channels 2, 4, 6, and 7. It is observed that stimulations on channels 4, 5, 6, 7, and 8 cannot be decoded from the LFP decoder at all, since the fine time information is averaged out in LFPs. For the spike trains decoder, the stimulation channels are not well discriminated. However, the combination of spike trains and LFPs enriched the stimulation information, which contributes to better discrimination of stimulation patterns among channels and also enables the model to capture the precise stimulation timing.

### 5.4. Open Loop Adaptive Inverse Control Results

The challenge of implementing a somatosensory prosthesis is to precisely control the neural response in order to mimic the neural response induced by natural stimulation. As discussed, the kernel-based adaptive inverse control diagram with tensor-product kernel is applied to address this problem. The adaptive inverse control model is based on a decoder which maps the neural activity in S1 to the microstimulation delivered in VPL. We proceed to show how the adaptive inverse control model can emulate the neural response to “natural touch” using optimized microstimulation.

In the same recording, open loop adaptive inverse control via optimized thalamic (VPL) microstimulations is implemented. First, the inverse controller *C*(*z*) is trained with 300 s of the data generated by recording the response to randomly patterned thalamic microstimulation. Then, the first 60 s of the neural response recorded during tactile stimulation at each touch site is used as the target pattern and control input. When this entire neural response sequence is fed offline to the controller, it generates a corresponding sequence of multichannel microstimulation amplitudes.

However, the generated microstimulation sequence needs further processing to meet the restrictions of bipolar microstimulation, before it applied to VPL. The restrictions and processing are the following.

1. The minimal interval between two stimuli 10 ms is suggested by the experimental setting. The mean shift algorithm [55] is used to locate the local maxima of a subsequence of stimuli (10 ms) for each single channel. The maximum amplitude and corresponding timing are used to set the amplitude and time of stimuli.
2. At any given time point, only a single pulse across all channels can be stimulated. Therefore, at each time point, only the maximum value across channels is selected for stimulation. The values at other channels are set to zero.
3. The maximum/minimum stimulation amplitude is set in the range [8 *μ*A–30 *μ*A], which has been suggested as the effective and safe amplitude range in previous experiments.

After this processing, the generated multichannel microstimulation sequence (60 s in duration) is ready to be applied to the microstimulator immediately following computation.

The neural response to the microstimulation is recorded and compared with the target natural response. Ideally, these two neural response sequences should be time-locked and be very similar. In particular, the portions of the controlled response in windows corresponding to a natural touch should match. As this corresponds to a* virtual touch* delivered by the optimized microstimulation, we define the term virtual touch to refer to the sequence of microstimulation patterns—the output of the controller—corresponding to a particular target natural touch.

**Figure 9:**
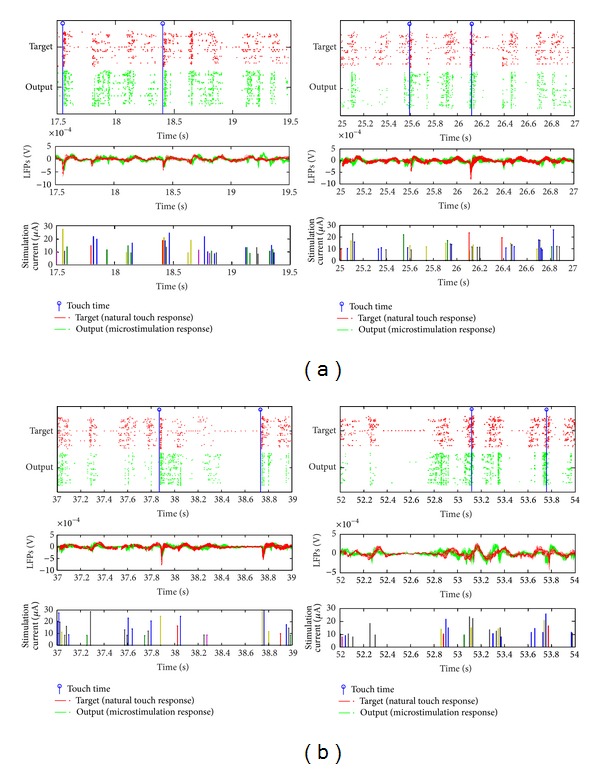
Neural responses to natural and virtual touches for touch on digit 1 (d1), along with the microstimulation corresponding to the virtual touches. Each of the four subfigures corresponds to a different segment of the continuous recording. In each subfigure, the timing of the touches, spatiotemporal pattern of spike trains and LFPs are shown in the top two panels; the bottom panel shows the spatiotemporal pattern of microstimulation, where different colors represent different microstimulation channels. The neural responses are time-locked, but not concurrently recorded, as the entire natural touch response is given as input to the controller which generates the optimized microstimulation patterns. When the optimized microstimulation is applied in the VPL, it generates S1 neural responses that qualitatively match the natural, that is, the target, response.

Portions of the neural response for both natural and virtual touches are shown in Figure 9. The responses are aligned to the same time scale, even though they were not recorded concurrently. It is clear that the multiunit neural responses recorded during the controlled microstimulation share similar spatiotemporal patterns as those in the target set. Each virtual touch is achieved by a sequence of microstimulation pulses that evokes synchronized bursting across the neurons. In addition, microstimulation pulses are delivered in between touch times to mimic population spiking that is not associated with the touch timing.

To evaluate performance, we concentrate on the following two aspects of virtual touches.

*(i) Touch Timing*. Whether the neural response to virtual touch is capable of capturing the timing information of the actual target touch is studied. 

*(ii) Touch Site*. Whether the actual target touch site information can be discriminately represented by neural activity controlled by the microcirculation is studied.

**Figure 10:**
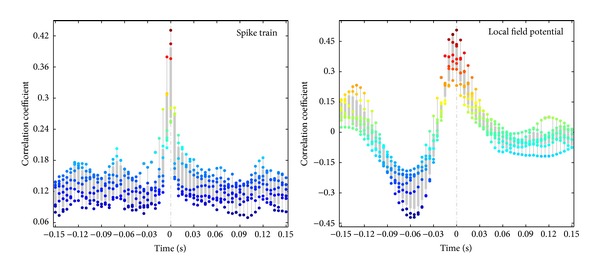
Correlation coefficients between the controlled neural system output and the corresponding target neural response stimulated by actual touch. Boxplot of correlation coefficients represents the results of 6 test trials. Each trial is corresponding to a particular touch site (digits: d1, d2, d4, p3, p1, and mp).

For* touch timing*, we estimate the correlation coefficients (CC) over time between virtual touch responses and the corresponding target natural touch responses. To simplify the spike train correlation estimation, we bin the data using 5 ms bins. The correlation coefficients of both spike trains and LFPs are calculated.  Figure 10 shows the boxplot plot of the correlation coefficients (CC) over 6 test trials, each of which corresponds to a particular natural touch site, a forepaw digit or pad (d1, d2, d4, p3, p1, or mp). It is observed that the maximum correlation coefficient is at lag zero for each trial, meaning that the virtual touch response is correctly time-locked. For each* touch site*, we estimate the similarity between the natural touch response and virtual touch response in the following two cases.

*(i) Matched Virtual*. Pairs consist of a virtual touch trial and a natural touch trial corresponding to the same touch site.

*(ii) Unmatched Virtual.* Pairs consist of a virtual touch trial and a natural touch trial corresponding to different touch sites.

We extract all the neural responses in the 300 ms window after touch onset and calculate the correlation coefficients between natural touch responses and virtual touch response across each pair of trials. The one-tailed Kolmogorov-Smirnov test (KS) is implemented to test the alternative hypothesis that the distribution of the correlation coefficients for the* matched virtual* case is higher than the distribution for the* unmatched virtual* case (the null hypothesis is that the distributions are the same). The correlation coefficients and *P* value of KS test for spike trains and LFPs are shown in Tables 2 and 3. The similarity between natural touch responses and virtual touch responses in the* unmatched virtual* case is found to be significantly lower than the* matched virtual* case for most touch sites (*P* value <0.05) except for touch site p3. Without psychophysical testing, it is unclear how effective the microstimulations are in producing true sensory sensations. Nonetheless, these are promising results to show the effectiveness of a controller utilizing the multiscale neural decoding methodology.

## 6. Conclusions

This work proposes a novel tensor-product-kernel-based machine learning framework, which provides a way to decode stimulation information from the spatiotemporal patterns of multiscale neural activity (e.g., spike trains and LFPs). It has been hypothesized that spike trains and LFPs contain complementary information that can enhance neural data decoding. However, a systematic approach to combine, in a single signal processing framework, these two distinct neural responses has remained elusive. The combination of positive definite kernels, which can be defined in both the spike train space and the LFP space, seems to be a very productive approach to achieve our goals. We have basically used two types of combination kernels to achieve the multiscale combination: sum kernels to “average” across different spike channels, as well as across LFP channels, which combine evidence for the neural event in each modality, and product kernels across the spike and LFP modalities to emphasize events that are represented in both multiscale modalities. The results show that this approach enhances the accuracy and robustness of neural decoding and control. However, this paper should be interpreted as a first step of a long process to optimize the joint information contained in spike trains and LFPs. The first question is to understand why this combination of sum and product kernels works. Our analyses show that the sum kernel (particularly for the spike trains) brings stability to the neural events because it decreases the variability of the spike responses to stimuli. On the other hand, the product kernel requires that the neural event presents at both scales to be useful for decoding, which improves specificity. If we look carefully at Figure 6, we can understand the effect of decoding with the product kernel. Notice that the correlation times of spikes and LFPs are very different (LFPs have a much longer correlation time). Moreover, composite kernel definition can be naturally configured to different brain areas and even neuronal types with distinctive firing patterns. Each pattern will lead to different correlation profiles, which will immediately tune the properties of the kernels across brain areas and neural populations. If only LFPs are used, we can expect that the response time of the decoder will be very long and miss some events. The product kernel in fact limits the duration of the LFP kernel to that of the spike kernel and brings stability to the spike kernel. This explains exactly the decoding results. Therefore, results show that the proposed tensor-product-kernel framework can effectively integrate the information from spikes and LFPs into the same model and enhance the neural decoding robustness and accuracy.

Furthermore, we applied the tensor-product-kernel framework in a more complex BMI scenario: how to emulate “natural touch” with microstimulation. Our preliminary results show that the kernel-based adaptive inverse control scheme employing tensor-product-kernel framework also achieves better optimization of the microstimulation than spikes and LFPs alone (results not shown). This result can be expected because the inverse controller is basically a decoder. However, we have to realize that not all the tasks of interest reduce to neural decoding, and we do not even know if neural control can be further improved by a different kernel design. This is where further research is necessary to optimize the joint kernels. For instance, we can weight both the channel information and the multiscale information to maximize the task performance using metric learning [40].

Overall, this tensor-product-kernel-based framework proposed in this work provides a general and practical framework to leverage heterogeneous neural activities in decoding and control scenario, which is not limited to spike trains and LFPs applications.

## Figures and Tables

**Algorithm 1:**
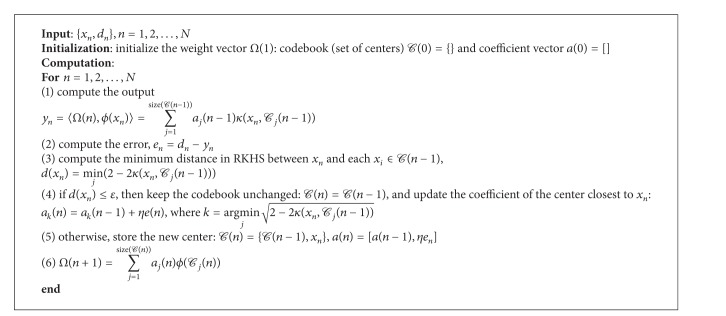
Quantized kernel least mean square (QKLMS) algorithm.

**Table 1:** Comparison among neural decoders.

Property	Input		
	LFP and spike	LFP	Spike
NMSE (mean/STD)	0.48/0.05	0.55/0.03	0.63/0.11

**Table 2:** Average and standard deviation of the correlation coefficient (CC) between natural touch spike train responses and virtual touch spike train responses (matched or unmatched). The *P* value is for the one-sided KS test between the matched and unmatched CC distributions.

Touch site	CC		
	Matched virtual	Unmatched virtual	*P* value
d1	0.42 ± 0.06	0.35 ± 0.06	0.00
d2	0.40 ± 0.05	0.37 ± 0.06	0.01
d4	0.40 ± 0.05	0.37 ± 0.05	0.02
p3	0.38 ± 0.05	0.37 ± 0.06	0.11
p2	0.40 ± 0.07	0.36 ± 0.05	0.00
mp	0.41 ± 0.07	0.37 ± 0.06	0.00

**Table 3:** Average and standard deviation of the correlation coefficient (CC) between natural touch LFP responses and virtual touch LFP responses (matched or unmatched). The *P* value is for the one-sided KS test between the matched and unmatched CC distributions.

Touch site	CC		
	Matched virtual	Unmatched virtual	*P* value
d1	0.42 ± 0.20	0.28 ± 0.23	0.00
d2	0.46 ± 0.13	0.28 ± 0.22	0.00
d4	0.41 ± 0.19	0.26 ± 0.21	0.00
p3	0.38 ± 0.18	0.29 ± 0.22	0.07
p2	0.33 ± 0.19	0.26 ± 0.23	0.20
mp	0.34 ± 0.17	0.25 ± 0.21	0.00
